# SinusCor: an advanced tool for heart rate variability analysis

**DOI:** 10.1186/s12938-017-0401-4

**Published:** 2017-09-18

**Authors:** Rhenan Bartels, Leonardo Neumamm, Tiago Peçanha, Alysson Roncally Silva Carvalho

**Affiliations:** 1 Pulmonary Engineering Laboratory, Biomedical Engineering Program, COPPE, Federal University of Rio de Janeiro, Cidade Universitária, Rio de Janeiro, Brazil; 2 School of Physical Education and Sport, University of São Paulo, São Paulo, Brazil; 3 Laboratory of Respiration Physiology, Carlos Chagas Filho Institute of Biophysics, Federal University of Rio de Janeiro, Cidade Universitária, Rio de Janeiro, Brazil

**Keywords:** Software, Heart rate variability, Time–frequency, Non-stationary signal

## Abstract

**Background:**

Heart rate variability (HRV) is a widespread non-invasive technique to assess cardiac autonomic function. Time and frequency domain analyses have been used in HRV studies, and their interpretations are linked with both clinical prognostic and diagnostic information. Statistical and geometrical parameters, Fast Fourier Transform and Autoregressive based periodograms are commonly used approaches for the assessment of stationary RR intervals (RRi) signals. However, some conditions result in non-stationary HRV behavior such as the “tilt test” and exercise. This study presents the SinusCor, a new free software for HRV analysis that includes the classical time and frequency domain indices and also techniques for non-stationary data analyses in both time (i.e. root mean squared of successive differences; RMSSD calculated with moving segments) and frequency domains (i.e. time–frequency analysis).

**Results:**

An example of RRi was acquired from a young male subject and its time and frequency domain indices were calculated. Time-varying and time–frequency analyses were also presented using the RMSSD and total power, respectively. Validation of the present software against a standard software for HRV analysis (Kubios v 3.0.1) was also performed [SinusCor vs. Kubios: RMSSD—93.96 (41.55) vs. 93.96 (41.55) ms; SDNN—101.29 (29.03) vs. 101.29 (29.03) ms; LF—50.42 (19.76) vs. 50.56 (19.56) n.u.; HF—49.57 (19.76) vs. 49.38 (19.56) n.u.; LF/HF—1.38 (1.08) vs. 1.38 (1.07)].

**Conclusions:**

SinusCor might be a useful tool for classical stationary and non-stationary HRV analysis.

## Background

Heart rate variability (HRV), defined as the variation of successive heartbeats (RR intervals, i.e. RRi), is a non-invasive tool for autonomic modulation assessment [1, 2]. Respiratory-coupled oscillations in heart rate (HR) are attributed to changes in parasympathetic activity over the sinoatrial node [2, 3], whereas lower frequency oscillations are imputed to phasic modifications in the sympathetic activity over the sinoatrial node [4] and also to other cardiovascular regulatory mechanisms [2, 5]. In general, a decrease in HRV is associated with poor cardiovascular health and prognosis [6, 7]. Classical HRV analyses are performed in stationary signals using time (statistical or geometrical analyses) or frequency (power spectral analysis) domains methods [2]. Most of the software used for HRV analysis employ these methods [8, 9], however the analysis of not well-behaved or non-stationary signals, such as those provided by “tilt test”—a clinical maneuver where the subject is submitted to an orthostatic stress; or exercise becomes laborious. To overcome this limitation, our study presents SinusCor, an user-friendly free software developed in Matlab (MathWorks, Massachusetts, USA), with a graphical interface that covers some functions included in others software, with additional features, such as time-varying and time–frequency analysis, different filters, windowing functions and R-peak detection algorithm for electrocardiogram (ECG) signals, with user’s manual edition.

## Implementation

### File extensions

This program supports inputs as RRi or ECG data with different file extensions: pure text (.txt), Polar^®^ heart rate monitor (.hrm), Suunto^®^ heart rate monitor (.sdf), Data Acquisition System (.bin) [10], Biopac^®^ (.acq) and Axon Binary File (.abf).

### R-peaks detection

**Fig. 1:**
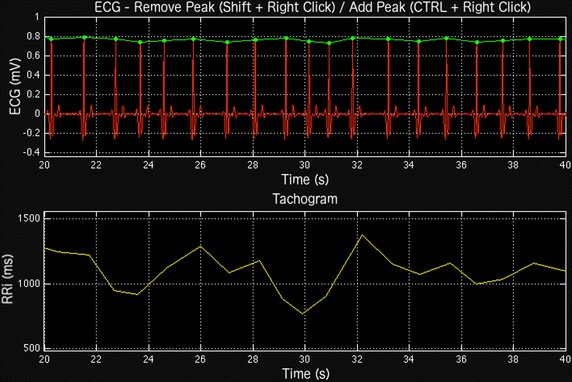
Example of an electrocardiogram (ECG) tracing with the R-peaks detected (upper panel) and the corresponding RR interval (RRi) series (lower panel)

SinusCor software uses a simple derivative-based method for R-peaks detection. Initially, the ECG signal is bandpass-filtered with a Butterworth filter with default passband of 5–200 Hz to remove baseline and high frequencies noises. It is possible to set different values for passband according to the specificity of each ECG signal. After filtering, the R peaks are detected if the first derivative of the filtered signal crosses zero and if that corresponding point of the ECG is greater than a fixed threshold, also supplied by the user. The fixed threshold algorithm may not detect correctly some R-peaks [11]. To deal with this problem, a manual edition, in which peaks can be added or removed, is available in our software. Figure 1 shows an example of an ECG tracing with the detected R-peaks and the corresponding RRi values.

## Filtering

During the acquisition process, artifacts or ectopic beats may contaminate the RRi series, affecting the HRV analysis [12]. RRi filtering is a widely used technique to remove non-natural beats from the tachogram [12–14]. Among existing filters, the present software counts with moving average, moving median and quotient filters [13]. The moving average consists on the convolution of the RRi signal with an N-length series of 1/N factors, in which N is the order of the filter. The moving-median filter applied to a given RRi value replaces this point by the median of the N (odd number) size interval centered on the current RRi value. The removal of non-natural beats using the quotient filter follows a simple rule: if the variation of two consecutive RRi values exceeds 20% (Eq. 1) the filter removes the ectopic beat. Quotient filter was implemented following the algorithm provided by Pikorski and Guzik [13]:

1 $$\begin{aligned} \frac{{RRi_{n} }}{{RRi_{n + 1} }} \ge 1.2\,\,or\,\,\frac{{RRi_{n} }}{{RRi_{n + 1} }} \le 0.8 \hfill \\ \frac{{RRi_{n + 1} }}{{RRi_{n} }} \ge 1.2\,\,or\,\frac{{RRi_{n + 1} }}{{RRi_{n} }} \le 0.8 \hfill \\ \end{aligned}$$

where RRi_n_ is the nth RRi value and RRi_n+1_ is the nth + 1 RRi value.

## Stationary signal HRV analysis

The following parameters can be calculated in SinusCor (Table 1).

**Table 1:** Heart rate variability parameters obtained from stationary analysis in SinusCor

Index	Description
Time domain	
RMSSD (ms)	Root mean squared of successive NN differences
SDNN (ms)	Standard deviation of NN intervals
pNN50 (%)	Percentage of successive NN differences greater than 50 ms
NN50	Count of successive NN differences greater than 50 ms
Mean RRi (ms)	Mean of RRi values
Mean HR (bpm)	Mean of HR values
Frequency domain	
Total power (ms^2^)	Energy of the PSD from 0 to 0.4 Hz (adjustable) in absolute values
VLF (ms^2^)	Energy of the PSD from 0 to 0.04 Hz (adjustable) in absolute values
LF (ms^2^)	Energy of the PSD from 0.04 to 0.15 Hz (adjustable) in absolute values
HF (ms^2^)	Energy of the PSD from 0.15 to 0.4 Hz (adjustable) in absolute values
LF/HF	Ratio of LF to HF
LFn.u	LF energy normalized by total power and VLF
HFn.u	HF energy normalized by total power and VLF

### Time domain methods

Time domain methods use statistical parameterizations of RR intervals. The Standard Deviation of the Normal RR intervals (SDNN) provides information on short and long-term variability of the signal (Eq. 2):

2 $$SDNN = \sqrt {\frac{1}{N - 1}\sum\nolimits_{j = 1}^{N} {\left( {RRi_{j} - \overline{RRi} } \right)^{2} } }$$

where N is the count of RRi values; RRi_j_ is the jth RRi value and $\overline{RRi}$ is the average value of the RRi series.

The root mean square of successive differences (RMSSD) represents the short-term variability (Eq. 3):

3 $$RMSSD = \sqrt {\frac{1}{N - 1}\sum\nolimits_{j = 1}^{N - 1} {\left( {RRi_{j + 1} - RRi_{j} } \right)^{2} } }$$

where N is the count of RRi values and RRi_j_ is the jth RRi value.

Also, the short-term (SD1) (Eq. 4) and long-term (SD2) (Eq. 5), measurements derived from a Poincaré plot, give similar information of RMSSD and SDNN, respectively, in terms of variability [15].

4 $$SD1 = \sqrt {2.SDNN^{2} - 2.SD2^{2} }$$

5 $$SD2 = \sqrt {2.SDNN^{2} - \frac{1}{2}.SDSD^{2} }$$

where SDNN is the standard deviation of the RRi value; SDSD is the standard deviation of the differences between adjacent RRi values.

The pNN50 quantifies the number of successive intervals differing more than 50 ms (nRRi_50_) divided by the total number of RR intervals (nRRi) and multiplied by 100.

6 $$pNN50 = \frac{{nRRi_{50} }}{nRRi}.100$$

Other simpler calculations are done by the software, such as the mean and the mean of successive differences of the RR intervals. Table 1 summarizes the indices calculated in time domain.

### Frequency domain methods

HRV analyses in frequency domain are performed through spectral decomposition of the RRi signal, using Fast Fourier Transform (FFT) or Autoregressive (AR) based methods and then decomposed into the following frequency components in absolute power values (ms^2^): very low frequency (VLF: 0.003–0.04 Hz), low frequency (LF: 0.04–0.15 Hz), high frequency (HF: 0.15–0.4 Hz) and total power (TP: 0–0.4 Hz) [2]. Indices derived from the spectral components such as the ratio of LF to HF (LF/HF), normalized LF (Eq. 7) and HF (n.u.) (Eq. 8) are also calculated. All frequency domain indices calculated in SinusCor are shown in Table 1.

7 $$LF_{{{\text{n}} . {\text{u}}}} = \frac{LF}{TP - VLF}.100$$

8 $$HF_{{{\text{n}} . {\text{u}}}} = \frac{HF}{TP - VLF}.100$$

In the SinusCor Software, it is possible to choose power spectral density (PSD) estimation through Welch’s periodogram [16] or via AR method. In Welch’s method, the frequency components are calculated using the average of the estimated PSD (Eq. 9) from P (Eq. 10) sub segments of the original RRi series [16].

9 $$PSD_{\left( f \right)} = \frac{1}{P}\sum\nolimits_{p = 1}^{P} {PSD^{\left( p \right)}_{\left( f \right)} }$$

where $PSD^{\left( p \right)}_{\left( f \right)}$ is the estimated PSD of each RRi series subsegment.

10 $$P = \left[ {\frac{N - D}{S}} \right] + 1$$

where N is the number of samples of the tachogram; D is the segment size and S is the shift between adjacent segments.

When the user chooses the AR, SinusCor estimates the PSD using the Burg algorithm. In this method, an AR prediction model, with the default order 16, fits the RRi series using the least square criteria. After the signal parametrization, the PSD is estimated using the frequency response of the model [17].

**Fig. 2:**
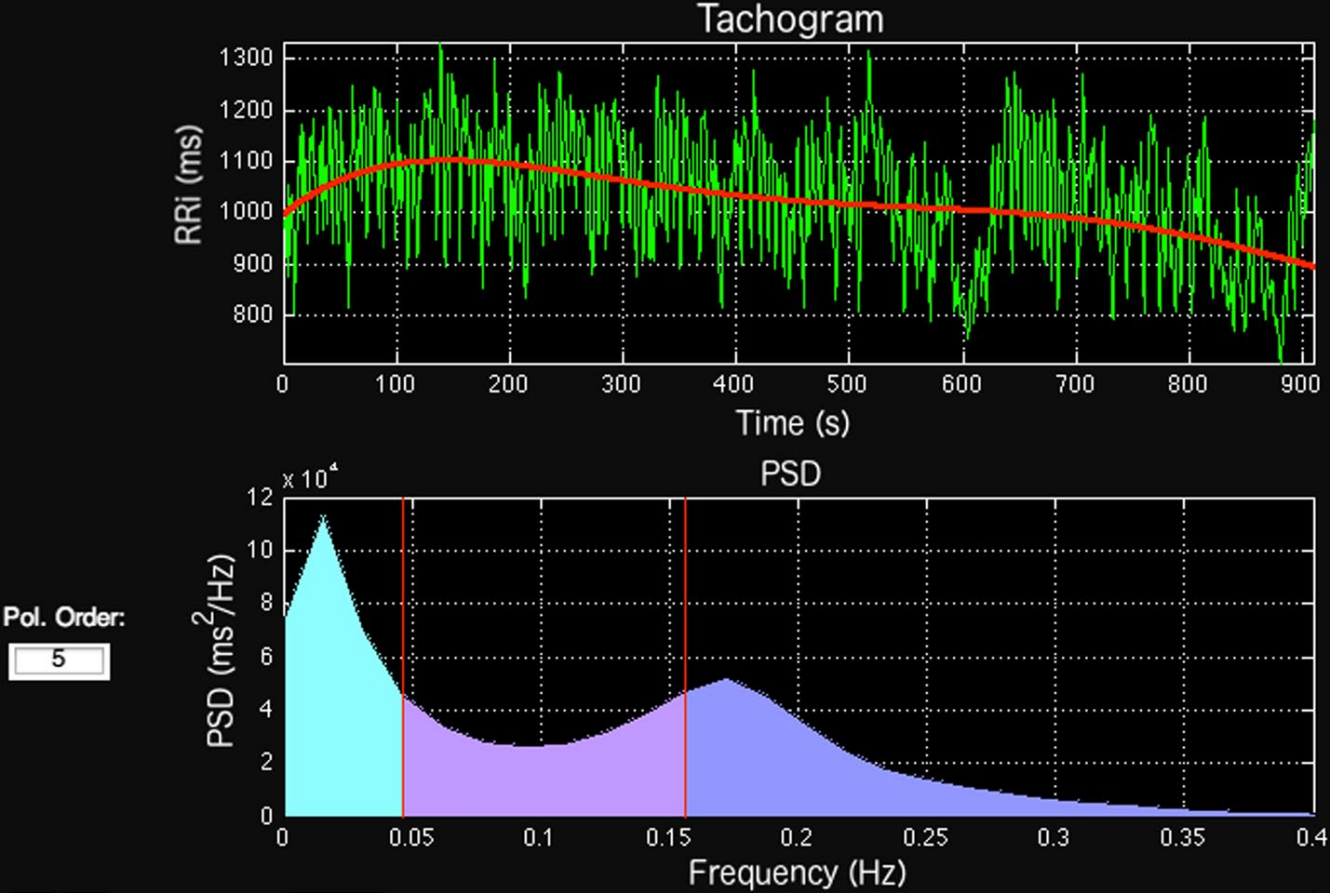
Example of a RR interval (RRi) series during rest period, detrended with a 5 degree polynomial (upper panel) and the corresponding power spectral density (PSD) function estimated with Welch’s method (lower panel)

Prior to the PSD estimation and frequency components calculation, the RRi series are interpolated and then resampled using a sampling frequency chosen by the user. SinusCor uses 4 Hz [2] as default value for resampling frequency, which can also be modified by the user. To reduce the influence of lower frequencies in the resulting PSD, it is possible to perform the detrending of the RRi series. The user can choose between linear (default), quadratic or cubic polynomials to be subtracted from the tachogram. It is also possible to choose a custom degree polynomial. In this case, the user chooses the polynomial degree, varying from 1 to N − 1, where N is the number of points in the tachogram. The superior panel of Fig. 2 presents an example of RRi series detrending using a polynomial with degree 5.

In the Welch’s method, the segment and overlap size can be adjusted. The default values are 256 and 128 (i.e. 50% of overlap), respectively. It’s also possible to apply different window functions (hanning, hamming, kaiser, blackman and triangular) to avoid spectral leakage. When AR method is chosen, the default value of the model order of 16 can be modified to fit different purposes. To achieve higher PSD (Fig. 2) resolutions, it is possible to use a zero-padding function. The area under the curve of each band can be calculated by the spectrum integration using trapezoidal approximation, with the frequency limits specified by the user. The adjustable frequency band makes the software useful for different applications [2, 18].

## Non-stationary signal HRV analysis

Since the RRi series may present non-stationary behavior, shorter segments can better represent the RRi changes over time [19–21]. SinusCor presents two methods for HRV analysis in non-stationary signals.

### Time-varying

**Fig. 3:**
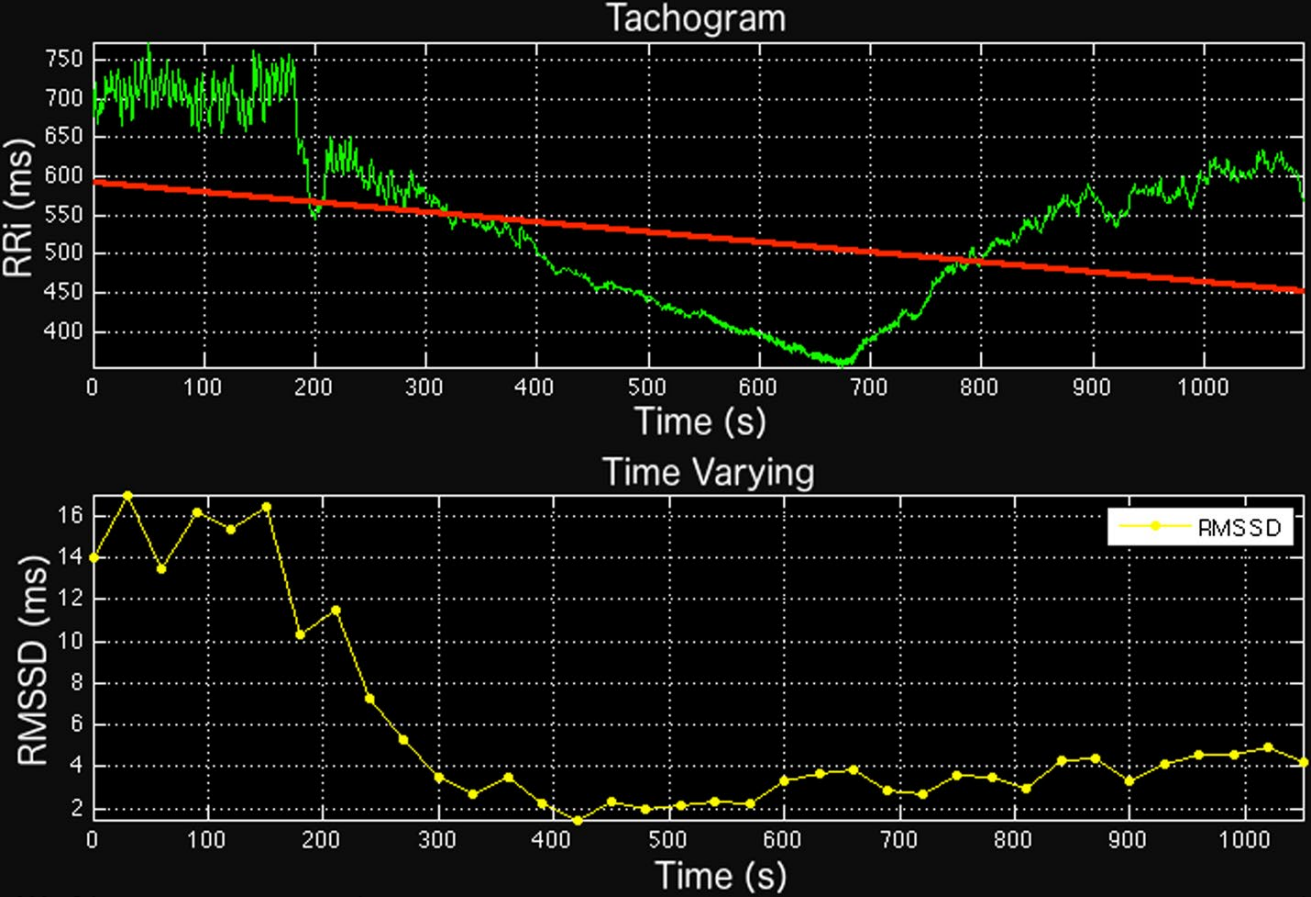
Non-stationary RR interval (RRi) series during an incremental exercise (upper panel) and the resulting RMSSD index calculated within 30 s segments without overlap (lower panel)

Time-varying analysis refers to the analysis of typical time domain indices of HRV in successive short segments [19, 21, 22]. In the SinusCor software, the user can set a segment and overlap size. All of the above listed time domain indices of HRV (Table 1) can be calculated using this tool. Figure 3 shows a typical example of RMSSD values over time with 30 s segments without overlap.

### Time–frequency

**Fig. 4:**
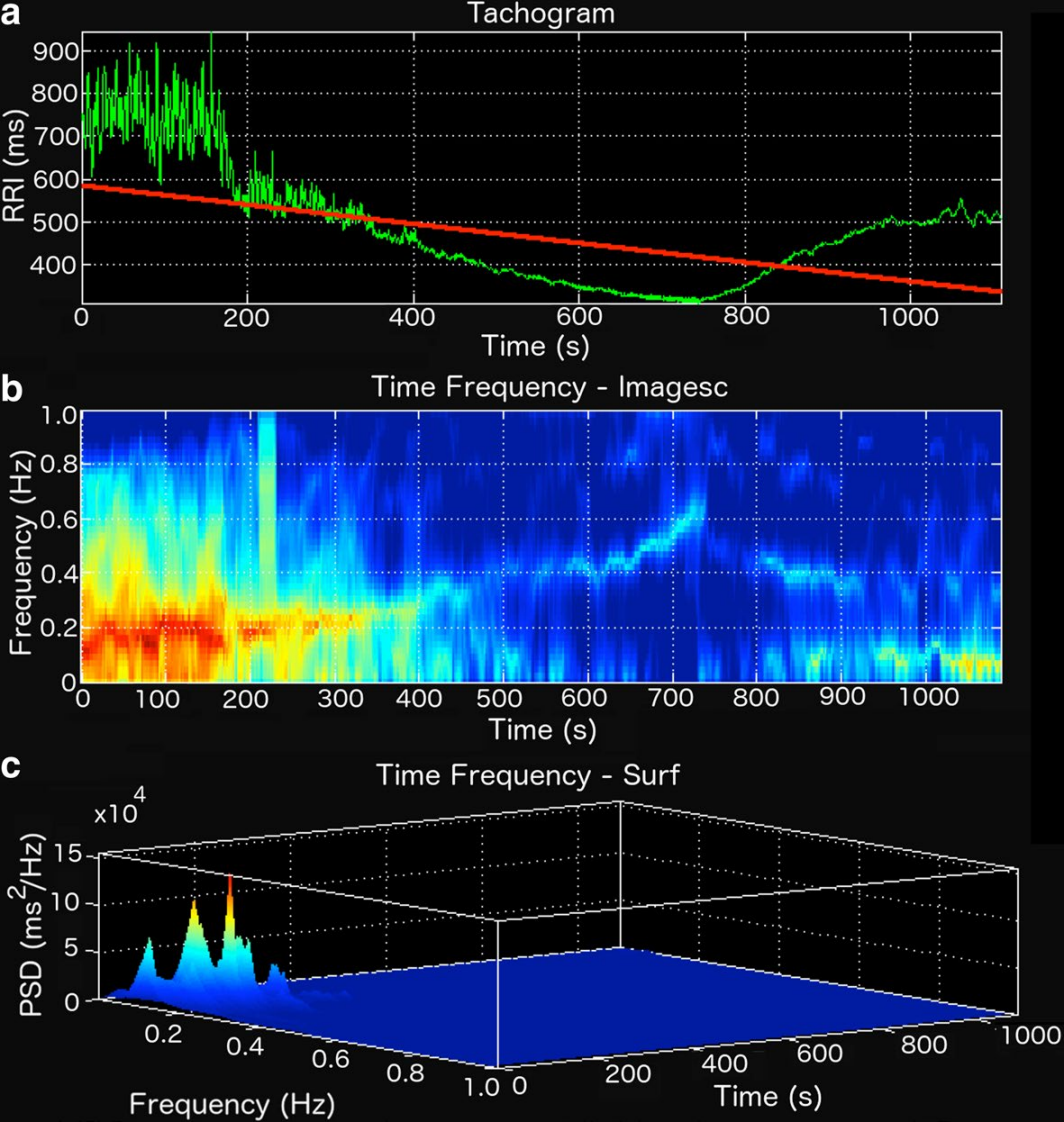
Non-stationary RR interval (RRi) series during a maximal effort exercise (**a**) and the resulting scaled colors map (**b**) and surface plot (**c**). *PSD* power spectral density

The time–frequency technique allows the estimation of spectral components of RRi time series with FFT- or AR-based methods over time [20, 23]. The algorithm works similar to the Welch’s method, but instead of calculating the average of all estimated PSD, the time–frequency method results in a 3D map (time, frequency and PSD) with the PSD for each sub-segments displayed over time (Fig. 4). Afterwards, the spectral components (Table 1) are calculated for each estimated PSD. The user may set the boundaries of each spectral component, the size of each RRi segment and the respective overlap size. AR method’s model order and the window function are also modifiable. For the sake of a better visualization of the spectrogram, in all methods a zero-padding option is also available. To visualize the resulting 3D map from the time–frequency analysis, the user can choose between a surface plot or an image with scaled colors (Fig. 4).

## Exporting results

**Fig. 5:**
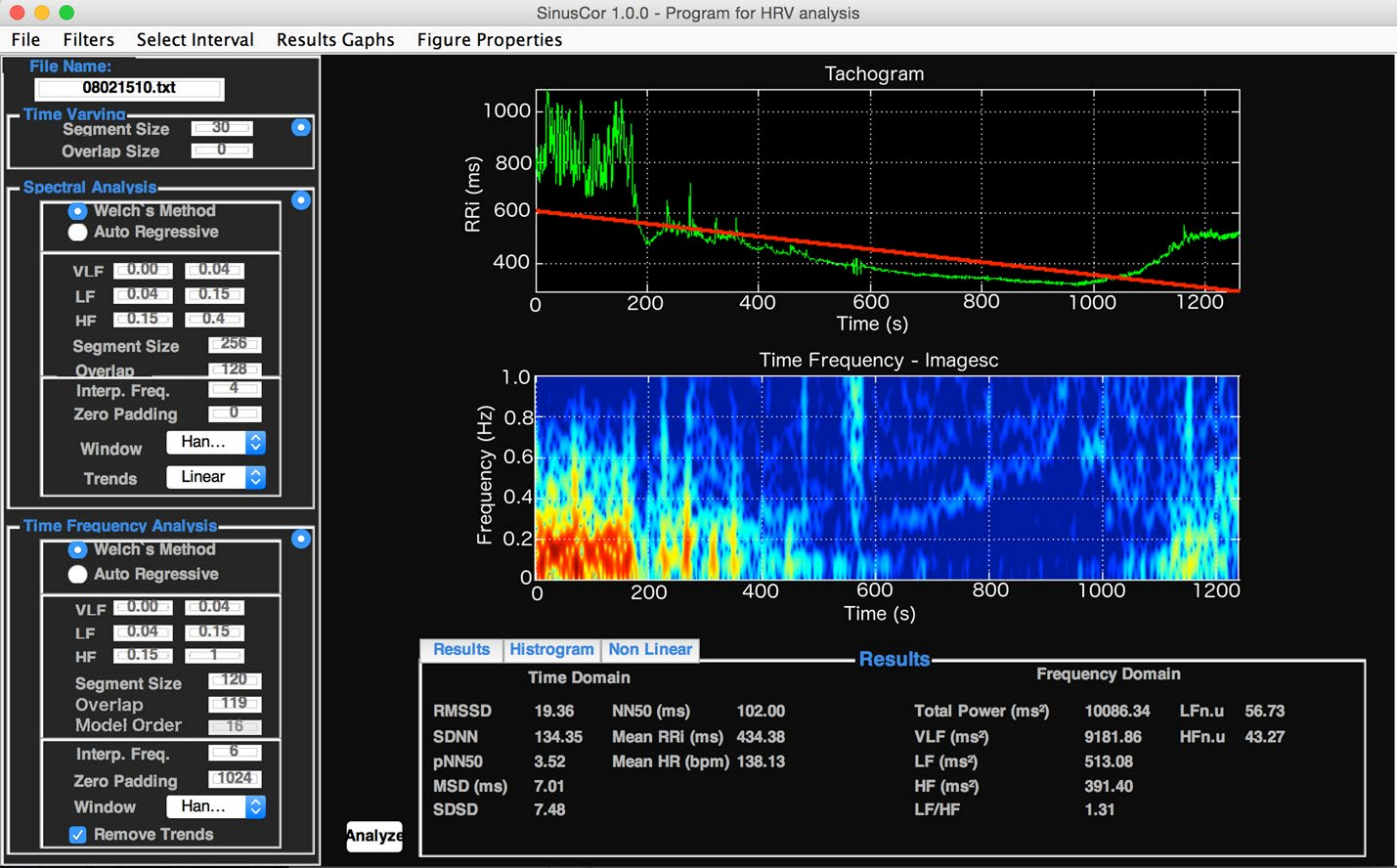
SinusCor interface with a non-stationary RR interval (RRi) series (upper panel) and the resulting time–frequency analysis represented as a scaled color map (middle and lower panels)

**Fig. 6:**
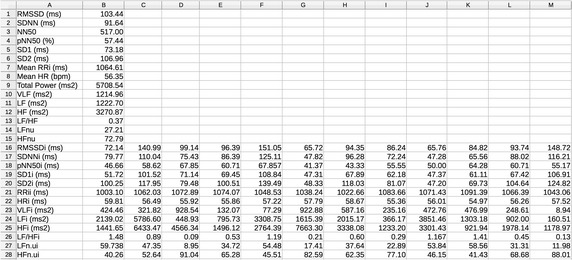
Exported.csv file containing the results from the analyses

**Fig. 7:**
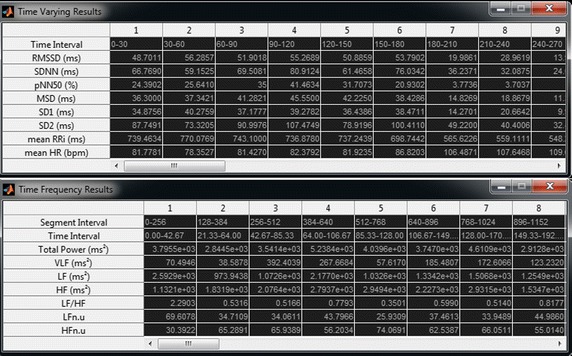
Tables containing the results from time-varying (upper panel) and from time–frequency (lower panel) analyses

The results from time and frequency domain analyses are displayed in the main interface, as shown in Fig. 5. Two options are available for accessing the results from the time-varying and time–frequency analyses: first, exporting all results to a.csv (comma-separated values) file as illustrated in Fig. 6 or; second, SinusCor provides two tables placed in different windows, one containing the results of the time-varying and the other containing the results of time–frequency analysis (Fig. 7).

## Results

### Analysis of stationary and non-stationary RRi series

To test the feasibility of SinusCor, we firstly analyzed HRV from a young male at rest and during an incremental exercise test in cycle ergometer (Ergo-Fit, Ergo Cycle 167, Pirmasens, Germany). For resting measurements, the subject remained 10 min in supine position, with spontaneous breathing. The exercise test was preceded by 3 min of pre-exercise measurements. The test workload was initially set to 25 W, and then was increased 25 W every minute until the subject’s exhaustion. Cycling was performed within a cadence of 55–65 rpm. After the test, the subject remained seated on cycle ergometer for 5 min in inactive recovery. RRi was monitored during the whole protocol using a heart rate monitor (PolarTM, S810, Finland; sampling frequency = 1000 Hz). After experimental session, data was imported into SinusCor for HRV analysis.

Resting HRV was calculated using conventional time and frequency domain analyses in the last 5 min of the 10-min resting. The exercise HRV was calculated using the time-varying and time–frequency analyses. For time-varying analysis, we calculated the RMSSD index for successive 30 s segments, without overlap, during the whole exercise test. Time–frequency analysis was estimated via Welch’s method in 512 samples segment with 50% overlap. The hanning window was applied and the signal was detrended using a linear polynomial fit.

**Fig. 8:**
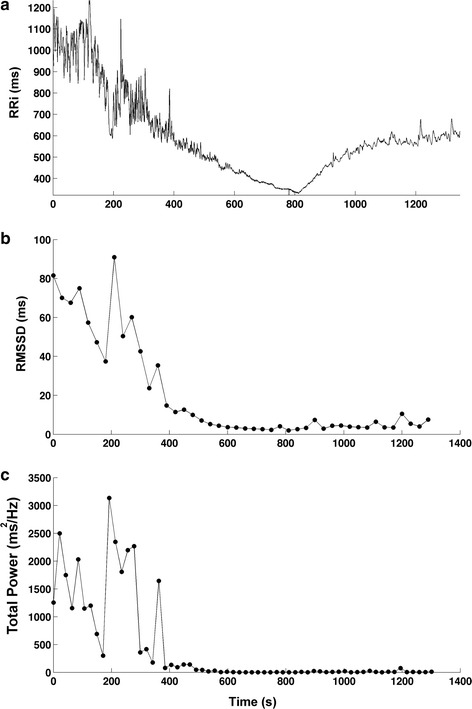
RR interval (RRi) series during a maximal incremental exercise and recovery (**a**) and the resulting RMSSD (**b**) and total power (**c**) from a healthy young male

Table 2 presents the resting HRV of the subject. Figure 8 shows the RRi series (panel a) and the resulting RMSSD (panel b) and total power (panel c) from the subject along the exercise test. At pre-exercise, the subject presented a mean RRi of 984.56 ms (corresponding to a heart rate of 61.70 bpm), a RMSSD of 66.41 ms, and a total power of 7300.87 ms^2^/Hz. Once the subject started the exercise, the RRi and HRV presented a progressive decrease, and, at the end of exercise, RRi was 338.34 ms (corresponding to a heart rate of 176.85 bpm), RMSSD was 2.95 ms, and total power was 3.79 ms^2^/Hz. During the recovery, RRi and HRV presented an exponential increase, and final RRi was 606.12 ms (corresponding a heart rate of 99.13 bpm), RMSSD was 7.53 ms, and total power was 14.73 ms^2^/Hz.

**Table 2:** Resting HRV indices of a healthy young male

Index	Value
Time domain	
RMSSD (ms)	73.10
SDNN (ms)	88.59
PNN50 (%)	47.99
NN50	489
Mean RRi (ms)	891.47
Mean HR (bpm)	67.30
Frequency domain	
Total power (ms^2^/Hz)	5499.53
LF (ms^2^/Hz)	1895.67
HF (ms^2^/Hz)	1616.05
LF/HF	1.17
LFn.u	53.98
HFn.u	46.02

### Validation against standard software

**Fig. 9:**
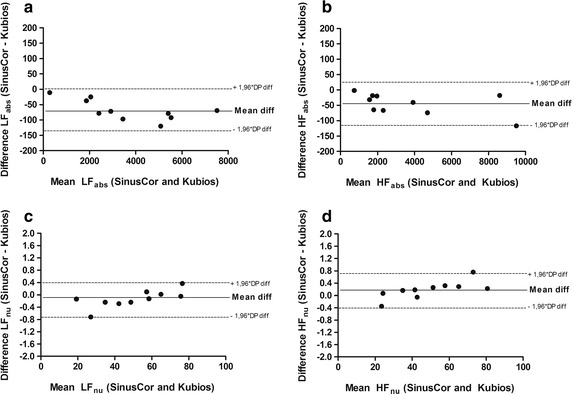
Bland–Altman plots (systematic bias ± limits of agreement) for **a** absolute LF. **b** Absolute HF. **c** Normalized LF; and **d** normalized HF

To validate the results provided by SinusCor, we used RRi signals from 10 healthy subjects during rest in the supine position during 900 s, and analyzed time and frequency domain HRV using SinusCor and Kubios (v 3.0.1, Biosignal Analysis and Medical Imaging Group, Finland), a widely used software for HRV assessment [9]. For time domain analysis, we performed calculations of RMSSD, SDNN, PNN50, NN50, mean RRi and mean HR. For frequency domain, we calculated total power, absolute and normalized LF and HF spectral bands, and LF/HF. In order to provide the same parameters for frequency domain analysis, in both software we used the Welch’s method of power spectrum estimation. In Kubios, the segment size was set to 300 s, with 50% overlap and 5 Hz sampling frequency. Once SinusCor offers segment size and overlap customization based on the number of samples instead of seconds, we chose a segment length of 1500 samples, 50% overlap, and 5 Hz resampling. For each segment, a hanning window was applied to avoid spectral leakage. Because the analysis considered the whole RRi series, five PSD estimation were averaged to obtain the final density function. The linear trend was removed in all signals in both programs, and no artifact correction nor any preprocessing method despite interpolation and resampling was applied to the signals. To assess the agreement between the software, the mean and standard deviation, mean difference and limits of agreement (LOA; 1.96 × standard deviation of differences) were calculated and shown in Table 3. For better visualization Fig. 9 shows a Bland–Altman [24] plot for LF, HF, LFn.u and HFn.u. No difference was found for time domain indices, whereas a slight tendency for bias was present in total power and absolute LF and HF indices on frequency domain, with lower values for SinusCor software.

**Table 3:** HRV indices difference and LOA provided by SinusCor and Kubios

Index	Mean ± SD SinusCor	Mean ± SD Kubios	Bias ± LOA
Time domain			
RMSSD (ms)	93.96 ± 41.55	93.96 ± 41.55	0.00 ± 0.00
SDNN (ms)	101.29 ± 29.03	101.29 ± 29.03	0.00 ± 0.00
PNN50 (%)	49.98 ± 18.32	49.98 ± 18.32	0.00 ± 0.00
NN50	448.30 ± 140.16	448.30 ± 140.16	0.00 ± 0.00
Mean RRi (ms)	1009.76 ± 112.98	1009.76 ± 112.98	0.00 ± 0.00
Mean HR (bpm)	60.13 ± 7.08	60.13 ± 7.08	0.00 ± 0.00
Frequency domain			
Total power (ms^2^/Hz)	9740.55 ± 4884.26	9850.13 ± 4921.08	−109.58 ± 136.42
LF (ms^2^/Hz)	3614.12 ± 2171.14	3682.07 ± 2.193.50	−67.95 ± 66.87
HF (ms^2^/Hz)	3661.61 ± 3056.75	3706.82 ± 3074.75	−45.21 ± 68.38
LF/HF	1.38 ± 1.08	1.38 ± 1.07	0.00 ± 0.00
LFn.u	50.42 ± 19.76	50.56 ± 19.56	−0.14 ± 0.55
HFn.u	49.57 ± 19.76	49.38 ± 19.56	0.19 ± 0.55

## Discussion

This study presented and validated the SinusCor software, a user-friendly and free software for HRV analysis that includes the classical time- and frequency domain indices and also techniques for non-stationary data analyses. To test the feasibility of our software, we analyzed resting and exercise HRV data of a young male. In this sense, all resting indices were within the normal range of HRV for healthy adults [25]. In addition, the progressive decrease in RRi and HRV during incremental exercise tests has been extensively demonstrated in literature [26–29]. These responses result from the typical autonomic responses occurring during exercise, i.e. a decrease in parasympathetic and an increase in sympathetic activity [30]. Likewise, the exponential increase in RRi and HRV at the recovery phase is an ordinary response and is attributed to the fast parasympathetic reactivation occurring at this phase [21]. Therefore, the results presented above are consistent with the expected physiological responses of the heart rate variability indices during and after exercise, which indicates the ability of SinusCor to capture such responses.

In order to perform a preliminary validation of SinusCor, we analyzed the agreement of resting time and frequency domain HRV indices between SinusCor and Kubios, a widely used software for HRV analysis [9]. When compared to Kubios, there were no differences in time domain indices, whereas the frequency domain indices presented small differences between software. Regarding the agreement analysis, the time domain indices presented a perfect agreement, whereas slight bias were found for absolute frequency domain indices, with lower values for SinusCor. The fact that time domain indices are calculated with simple statistical equations and do not depend on preprocessing techniques nor on different methods for estimation can explain these findings. Although we provided the same parameters for frequency domain analysis in both software, Kubios does not inform some settings parameters that can lead to slight differences in frequency domain indices, such as Window function and integration method. Despite that, it is worth mentioning that differences in frequency domain indices were clearly small compared to the magnitude of the indices (i.e. 1–2%) and, interestingly, when the indices were exposed in normalized units (LFn.u, HFn.u and LF/HF), these differences and bias were not present anymore. All these results together indicate that SinusCor presents reliable results for both time and frequency domain analysis of resting HRV.

The present paper describes a software for advanced HRV analysis developed in Matlab. SinusCor allows the calculation of the most common indices of HRV in both time and frequency domains for stationary RRi data. It is also possible to analyze non-stationary HRV signals using time-varying analysis, which allows the calculation of classical time domain indices in RRi signals with amplitude and frequency variations; and time–frequency analysis, for spectral decomposition over time, using FFT or AR based methods. Time-varying and time–frequency analysis can be useful for researchers working with non-stationary HRV signals, such as the exercise or other physiological maneuvers.

SinusCor was developed to meet the demand of physiologists working with HRV in both clinical and research fields. This software allows the user to perform the most common and clinically validated analyses of stationary and non-stationary HRV analyses [21]. The graphical interface offers a user-friendly experience which makes parameter changing intuitive and easy for users. For conventional time and frequency domain analyses, SinusCor provides all of the preprocessing customization required in most of the guidelines for HRV analysis. For non-stationary time-dependent analyses, SinusCor provides options for specific customization, including setting the segment and overlap sizes and adjusting the spectral boundaries. Furthermore, SinusCor allows the separated visualization of the results of time-varying and time–frequency analyses, which makes the user interpretation clearer and easier. These features are not provided by any other software, therefore limiting the HRV evaluation in situations such as physical exercise. All figures that SinusCor generates can be exported to be embedded in scientific papers or patient reports. The results can also be exported as .csv tables or in separated windows for later analyses.

This paper herein presents the first version of SinusCor. Many other features will be implemented in the future based on the users’ feedback and suggestions. SinusCor is freely available for both academic and clinical use and can be downloaded on http://bit.ly/sinuscor, where tutorial videos and example files can also be found in order to help users.

## Conclusion

SinusCor is a useful tool for classical heart rate variability analysis and for RRi signals with non-stationary behavior. New versions of will continue to be developed offering new features and better performance.

## Abbreviations

HRV: heart rate variability
HR: hear rate
ECG: electrocardiogram
FFT: Fast Fourier Transform
AR: Autoregressive
RRi: RR intervals
RMSSD: root mean square of successive differences
SDNN: standard deviation of normal RR intervals
SD1: short-term beat-to-beat R–R variability from the Poincaré plot
SD2: long-term beat-to-beat R–R variability from the Poincaré plot
pNN50: percentage of successive NN differences greater than 50 ms
NN50: count of successive NN differences greater than 50 ms
VLF: very low frequency
LF: low frequency
HF: high frequency
TP: total power
PSD: power spectral density
LFn.u: low frequency in normalized units
HFn.u: high frequency in normalized units
csv: comma-separated values
LOA: limits of agreement
